# A Case of Mucinous Adenocarcinoma in a Urethral Diverticulum

**DOI:** 10.7759/cureus.95570

**Published:** 2025-10-28

**Authors:** Ryan P Lawson

**Affiliations:** 1 Diagnostic Radiology, Medical University of South Carolina, Charleston, USA

**Keywords:** female urethral diverticulum, gu radiology, malignant transformation, mucinus adenocarcinoma, pelvic cystic lesion

## Abstract

Urethral diverticula are uncommon observations encountered in the female pelvis and arise from the posterolateral wall of the urethra. While the pathogenesis of urethral diverticula is unsettled, they are presumably multifactorial, and several theories are addressed in this case report. Various complications are associated with urethral diverticula; however, the feared association is malignant transformation into a diverticular neoplasm.

This case highlights a unique instance of a urethral diverticulum complicated by mucinous adenocarcinoma, with intestinal features, in a female patient with underlying human immunodeficiency virus (HIV), which contributes to the limited literature on the intersection of urethral diverticula, immunocompromised states, the importance of multimodality imaging, and therapeutic options in the unique patient population.

## Introduction

Urethral diverticula are rare observations that occur more frequently in women than men [1]. Defined as a focal outpouching of the urethra, they most commonly arise from the posterolateral wall of the middle third of the urethra [2]. Approximately 0.6-6% of women will develop urethral diverticula, most frequently occurring in the third to sixth decades of life [3-5]. The pathogenesis of urethral diverticula is uncertain and likely multifactorial. Among the more widely accepted etiologies are chronic inflammation and obstruction of paraurethral glands [6]. There are a variety of complications associated with urethral diverticula, which include recurrent infection, urinary incontinence, calculus formation (approximately 1.5-10%), and the development of intradiverticular neoplasms such as mucinous adenocarcinoma [5,7]. Mucinous adenocarcinoma is a type of cancer that arises from glandular tissue and is rarely found in a urethral diverticulum. This case report presents a case of mucinous adenocarcinoma arising within a urethral diverticulum and highlights the importance of imaging in the diagnosis as well as management of this condition.

## Case presentation

**Figure 1:**
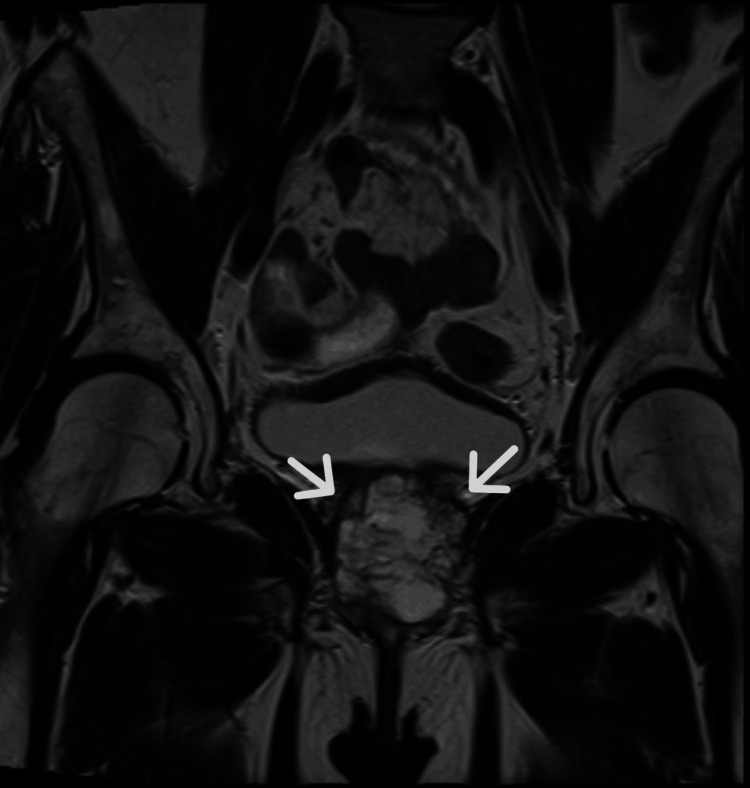
Coronal T2-weighted magnetic resonance image of a complex, multilobulated cystic periurethral mass denoted by the white arrows Often, this entity is described as "saddlebag" in appearance with relation to the urethra.

**Figure 2:**
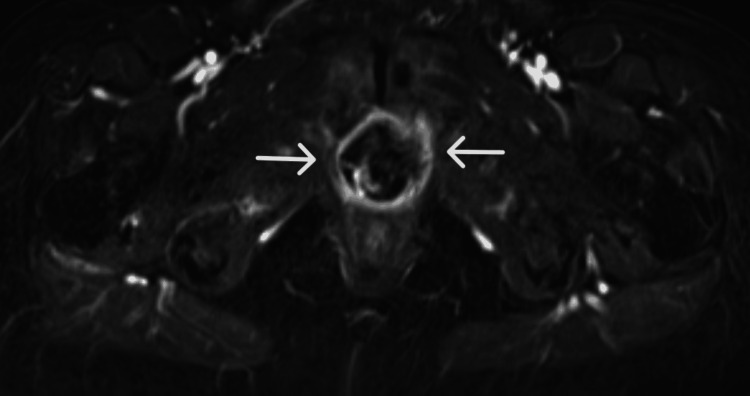
Axial T1-weighted postcontrast subtraction magnetic resonance image of a periurethral mass demonstrating peripheral and solid nodular central enhancement, denoted by the white arrows

**Figure 3:**
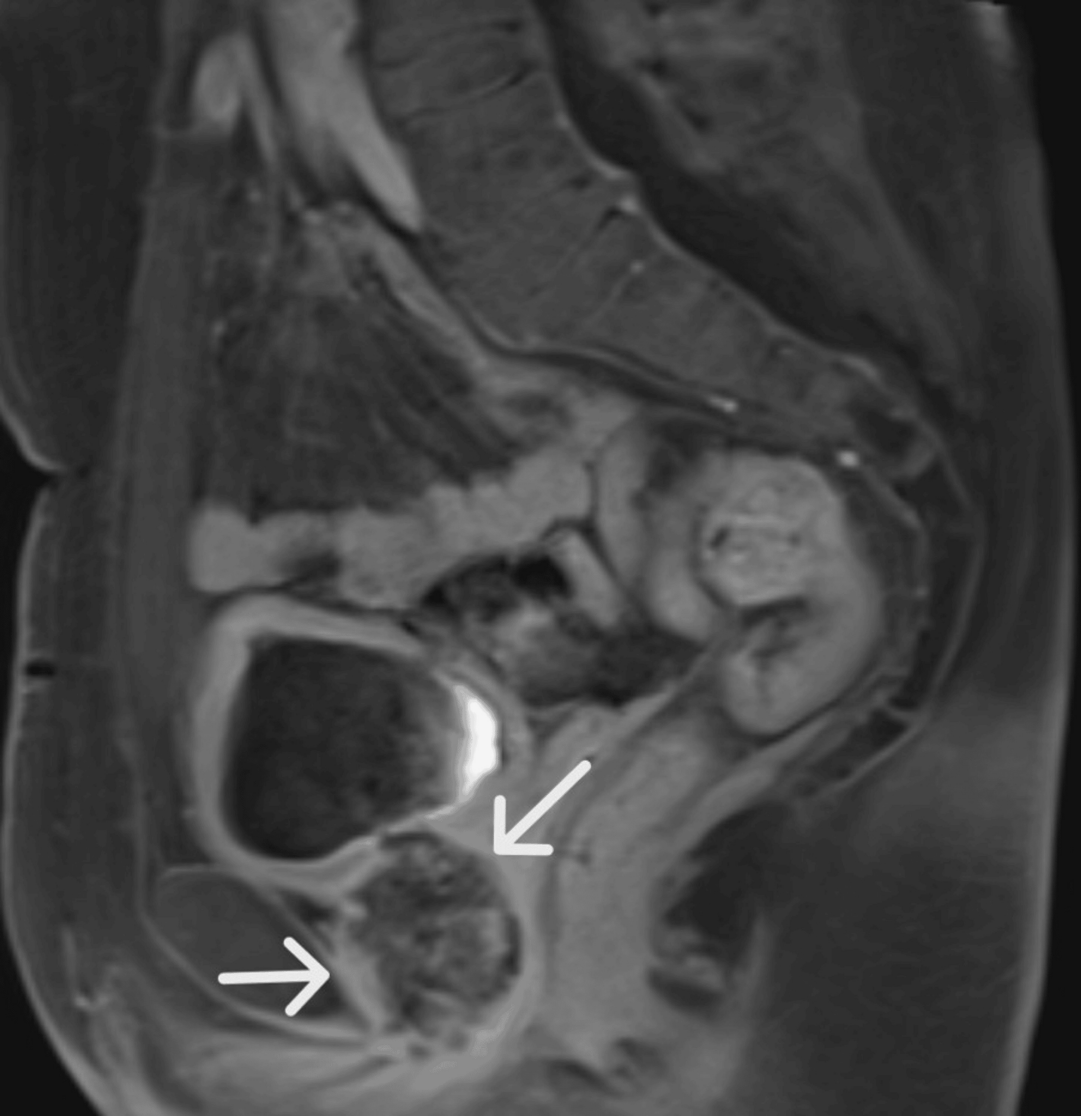
Sagittal T1-weighted postcontrast magnetic resonance image of a heterogeneously enhancing periurethral mass just beneath the urinary bladder, denoted by the white arrows

**Figure 4:**
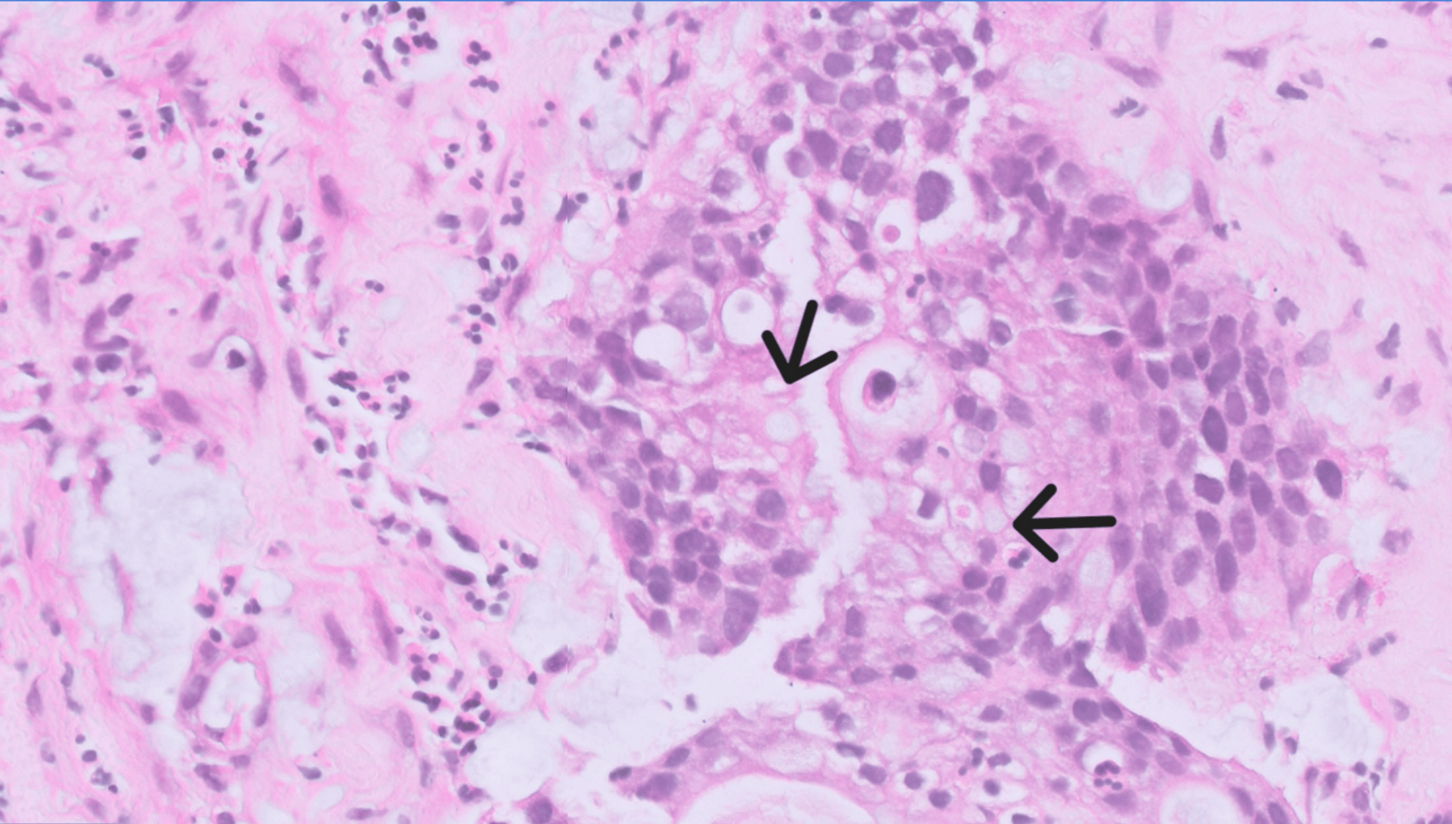
Mucinous type adenocarcinoma from a transvaginal urethral biopsy of the periurethral mass Hematoxylin and Eosin (H&E), 40X. Black arrows denote intra and extracellular mucin.

A 71-year-old African American female with a past medical history of human immunodeficiency virus (HIV) presented to an emergency department with complaints of progressively worsening urinary retention for the past 2 months. Physical exam was positive for suprapubic tenderness, but otherwise unremarkable. Routine hematologic and biochemical analysis revealed evidence of acute kidney injury. Otherwise, laboratory analysis was within normal limits. A bedside sonographic bladder scan was performed, demonstrating a volume of over 1000 mL. Initial attempts to place a 12 French Foley catheter in the emergency department were unsuccessful; therefore, urology was consulted for further management. Ultimately, the patient underwent cystourethroscopy, revealing a urethral diverticulum and an associated obstructing mass. Magnetic resonance (MRI) imaging of the pelvis was subsequently performed and revealed a 4.2 x 3.7 cm heterogeneously enhancing preiurethral cystic mass with extension into the prevesicular space, base of the bladder, and pelvic floor musculature (Figures 1-3). There was also a mass effect upon the anterior wall of the vagina with indeterminate extension of disease. A computed tomography (CT) scan of the abdomen and pelvis performed for the purposes of staging revealed enhancing peritoneal nodularity with correlative hypermetabolic activity on fluorodeoxyglucose positron emission tomography/computed tomography (FDG-PET/CT). For the purpose of tissue diagnosis, a transvaginal biopsy was performed. Histologically, the biopsy samples demonstrated metaplastic mucin-filled goblet cells intermixed with normal urothelium (Figure 4). Immunohistochemical stains, such as CDX-2, PAX-8, CK7, and CK20, can be utilized for further characterization; however, they were deemed unnecessary in this case [8]. With regard to treatment, the patient underwent neoadjuvant chemoradiation prior to radical cystectomy, ileal conduit formation, urethrectomy, anterior vaginectomy, and pelvic lymphadenectomy. Of importance for this particular clinical presentation, the patient's HIV was well-controlled on highly active antiretroviral therapy (HAART) with undetectable viral loads on Odefsey, which permitted the utilization of capecitabine for neoadjuvant chemotherapy to improve surgical outcome. The final surgical pathologic staging was ypT1 ypN0, given invasion limited to subepithelial connective tissue and the lack of nodal involvement. On post-treatment surveillance imaging, this case was complicated by osteomyelitis of the pubic symphysis managed with a six-week course of outpatient IV Zosyn followed by oral Doxycycline and Augmentin.

## Discussion

The differential diagnosis of urethral diverticular malignancy includes transitional cell carcinoma, squamous cell carcinoma, and adenocarcinoma, with adenocarcinoma representing the most frequent histologic subtype, accounting for approximately 60% of cases [9]. Among these, mucinous adenocarcinoma is particularly rare, defined by extracellular mucin comprising more than 50% of the tumor volume and generally associated with a worse prognosis than conventional adenocarcinoma [10]. Its occurrence within a urethral diverticulum is exceedingly uncommon, with a reported incidence of 3-6% among diverticular adenocarcinomas [11,12] and an estimated 0.01% among all female genitourinary malignancies [13].

Diagnosis of urethral diverticular carcinoma remains challenging due to its non-specific and often insidious symptomatology. Common presentations, such as urinary retention, dysuria, or recurrent urinary tract infections, can mimic benign conditions, often resulting in delayed diagnosis. In this case, the patient presented with a two-month history of progressively worsening urinary retention, yet imaging revealed a significant mass at diagnosis. This discrepancy suggests a period of subclinical tumor growth that may have been masked by non-specific symptoms or attributed to underlying comorbidities such as HIV-related bladder dysfunction or medication effects. Similar diagnostic delays have been reported in the literature, where symptoms often precede diagnosis by several months, emphasizing the need for a high index of suspicion when evaluating persistent lower urinary tract symptoms in women [14].

Historically, voiding cystourethrography (VCUG) was the preferred diagnostic modality, with a diagnostic accuracy of approximately 85% [15]. However, magnetic resonance imaging (MRI) has largely supplanted VCUG due to its superior soft-tissue characterization and ability to delineate the extent of disease. In the present case, MRI was instrumental not only in identifying the periurethral cystic lesion consistent with a diverticulum but also in detecting the enhancing solid intradiverticular component, which prompted biopsy and confirmed malignancy. This underscores MRI’s critical role in both detection and staging, as well as in guiding management decisions.

Although CT of the chest, abdomen, and pelvis is a more standard imaging modality for staging urethral diverticular carcinoma, PET/CT was also performed in this case presentation. While not routinely indicated, several case reports and small series have suggested that PET/CT may provide additional sensitivity in detecting nodal or distant metastases in rare urethral and bladder malignancies, particularly in high-grade or aggressive histologic subtypes [16]. Its use in this case may also reflect the availability of advanced imaging at an academic tertiary care center, allowing for more comprehensive staging and potentially guiding multidisciplinary treatment planning.

The patient’s final pathologic stage was ypT1 ypN0, indicating a tumor confined to the subepithelial connective tissue with no nodal involvement. This staging reflects a favorable response to preoperative neoadjuvant therapy and likely down-staging from an initially more advanced disease based on imaging appearance. Neoadjuvant chemotherapy was selected, given the aggressive histologic subtype, potential for micrometastatic disease, and favorable patient activity status--a strategy supported by limited case series and extrapolated from treatment paradigms for urethral and bladder adenocarcinomas [17]. There is no consensus in the literature regarding the management of diverticular malignancy. With that said, management options for urethral diverticular malignancy range from local excision to anterior pelvic exenteration, depending on the tumor’s extent and invasion of adjacent structures. In this case, the treatment plan balanced disease control with functional preservation, informed by the patient’s well-controlled HIV status, which permitted tolerance of multimodal therapy.

Compared to previously reported cases, this patient’s presentation was notable for the short duration of symptoms yet substantial tumor burden at diagnosis, suggesting either rapid tumor growth or masked symptomatology. This highlights a key pitfall in managing periurethral pathology--delayed recognition of malignancy within a presumed benign diverticulum. Moreover, patients with chronic comorbid conditions such as HIV may present with atypical symptom patterns or clinician anchoring bias, further contributing to diagnostic delay. Early use of pelvic MRI in patients with persistent urinary retention or recurrent infections could mitigate such delays and facilitate earlier intervention.

This case illustrates the diagnostic complexity and aggressive nature of mucinous adenocarcinoma arising within a urethral diverticulum. Timely diagnosis requires a high index of suspicion and early utilization of advanced imaging. Management should be individualized, incorporating multimodal therapy and multidisciplinary input to optimize oncologic outcomes while considering the patient’s comorbidities and functional status.

## Conclusions

This case demonstrates that mucinous adenocarcinoma arising from a urethral diverticulum, while rare, can present with subtle or rapidly progressive lower urinary tract symptoms that may delay diagnosis. Advanced imaging, particularly MRI and CT, are essential for accurate lesion characterization, staging, and treatment planning. Multimodal therapy, including neoadjuvant chemotherapy and surgical excision, may be appropriate for aggressive histologic subtypes and patients with good performance status. Clinicians should maintain a low threshold for considering malignancy in women with persistent or atypical urinary symptoms, and thorough evaluation of female pelvic anatomy with targeted imaging can facilitate early detection and improved outcomes.
